# Moderate temperature reduction is sufficient for prevention of 5-fluorouracil-induced oral mucositis: an experimental in vivo study in rats

**DOI:** 10.1007/s00280-022-04495-3

**Published:** 2022-12-01

**Authors:** J. Walladbegi, M. Dankis, Ö. Aydogdu, M. Jontell, M. Winder

**Affiliations:** 1 Department of Oral Medicine and Pathology, Institute of Odontology, The Sahlgrenska Academy, University of Gothenburg, PO Box 450, 405 30 Gothenburg, Sweden; 2 Department of Pharmacology, The Institute of Neuroscience and Physiology, The Sahlgrenska Academy, University of Gothenburg, Gothenburg, Sweden

**Keywords:** Animal model, Chemotherapy, Cryotherapy, IL-6, TNF-α

## Abstract

**Purpose:**

The current idea of how oral mucositis (OM) develops is primarily based on hypotheses and the early events which precede clinically established OM remain to be demonstrated. Cryotherapy (CT) continues to have considerable promise in clinical settings to reduce chemotherapy-induced OM. Although being effective, the knowledge is scarce regarding the ideal temperature for prevention of OM. Thus, the present study had two main objectives: (i) to develop an animal model to investigate the early events of OM; (ii) to study at what cooling temperature these early events could be abolished.

**Methods:**

Male Sprague–Dawley rats were anaesthetized and given an intravenous bolus dose with the cytostatic drug fluorouracil (5-FU). During the first hour following injection with 5-FU, the oral cavity of the rats was cooled to a mucosal temperature at the range of 15–30 ^○^C, or left uncooled (35 ^○^C), serving as control. After 3–5 days, the rats were euthanized, and the buccal mucosa was excised. Subsequently, mucosal thickness and expression of IL-6 and TNF-α were analyzed with immunohistochemistry and enzyme-linked immunosorbent assay (ELISA).

**Results:**

Five days following treatment with 5-FU, a statistically significant thickening of the oral mucosa occurred, and a distinct expression of both IL-6 and TNF-α were observed. The cryo-treated groups (15–30 °C) displayed statistically significantly thinner mucosa as compared to the control group (35 °C). The ELISA showed an increase in expression of the proinflammatory cytokines IL-6 and TNF-α in tissues exposed to 5-FU that were treated with increasing temperatures (15–30 °C).

**Conclusion:**

Bolus *i.v.* injection with 5-FU in rats can be used to create a functional animal model for chemotherapy-induced OM. Further, moderate temperature reduction is sufficient to reduce the early events which may precede clinically established OM.

## Introduction

Oral mucositis (OM) is defined as the inflammation of the mucous membrane lining the oral cavity. Once established clinically it can give rise to a number of symptoms, including pain, dysphagia, and taste alterations [1, 2]. Furthermore, OM can severely impair quality of life [3] and in a subset of patients act as a port of entry for systemic infection, leading to sepsis and death [4]. There are several suggested risk factors that could influence the course and severity of OM. The prime example is chemotherapy, i.e., antineoplastic treatment, currently used for a variety of malignant diseases to reduce tumor cell growth or even instigate cancer remission [5]. As these agents act systemically and lack the ability to distinguish between rapidly dividing healthy cells and their malignant counterparts, it is impossible to effectively treat the cancer without exposing the patient to various adverse effects, e.g., OM [6]. While the cytotoxicity of chemotherapy is a well-established risk factor for OM, the exact tissue reaction that occurs prior to OM is yet to be elucidated

The generally accepted model for development of OM is thought to encompass several complex subepithelial events, resulting in epithelial basal cell injury, either directly through DNA damage or indirectly by reactive oxygen species. This damage initiates in turn a complex series of events that involve enhanced enzymatic activity and activation of several transcription factors. Activation of NF-κB, which is the most studied pathway regarding mucositis development, drives the upregulation of genes that encode for several proinflammatory cytokines, e.g., interleukin-6 (IL-6) and tumor necrosis factor alpha (TNF-α). These two have demonstrated activities in the pathogenesis that precedes clinically established OM [7]. However, the current understanding of how OM develops is primarily based on hypotheses and several steps in the pathobiology remain to be conclusively demonstrated. In particular, this pertains to early events that precede clinically established OM.

Cryotherapy (CT), i.e., the use of ice or cooling devices to cool the oral mucosa, continues to have considerable promise in clinical settings to reduce chemotherapy-induced OM [8–12]. However, while CT effectively alleviates OM in patients receiving chemotherapy, the knowledge is scarce regarding the ideal temperature for prevention of OM. In fact, intraoral mucosal temperatures following cooling have only been conducted in two previous studies, both from our group [13, 14]. The first study showed a mean temperature reduction of ~ 12 °C (from 36 °C to 24 °C), and the second study ~ 8 °C (from 36 °C to 28 °C), following one hour of cooling with crushed ice. However, in a more recent in vitro study, we demonstrated that an artificial oral mucosa, preincubated at 20 °C were better preserved following exposure to the antineoplastic drug 5-fluorouracil (5-FU), as compared to models preincubated at higher temperatures [15]. Thus, it is of interest to pinpoint the ideal temperature to prevent OM in an in vivo setting.

The present study had two main objectives. The first was to develop a robust animal model to investigate the early events which precedes OM. The second was to establish the highest temperature at which these early events could be abolished.

## Materials and methods

### Ethical approval

The study was approved by the local Animal Ethics Committee at the University of Gothenburg, Sweden (permit #1603/2018). All experiments were designed to minimize the suffering of the animals during the experimental procedures.

### Materials

All substances and materials were purchased from Sigma-Aldrich (St. Louis, MO, USA) unless otherwise stated.

#### Surgical procedures

Medetomidine (Domitor Vet.; Orion Pharma, Espoo, Finland), ketamine (Ketalar; Pfizer, New York, USA), pentobarbital (60 mg/mL; APL, Gothenburg, Sweden), buprenorphine (Bupaq Multidose vet.; Salfarm Scandinavia, Helsingborg, Sweden), 5-fluorouracil (5-FU; Fluorouracil Accord; Accord Healthcare AB, Solna, Sweden), saline (Natriumklorid B. Braun; 9 mg/mL; Braun, Melsungen, Germany).

#### Immunohistochemistry (IHC)

Prolong gold antifade reagent with DAPI (Invitrogen, Burlington, Canada), PBS tablets (Invitrogen), ethanol (99.5%; Kemetyl, Stockholm, Sweden), xylene (Kemetyl), normal goat serum (Vector Laboratories, Burlingame, Canada), Triton X-100, sucrose, NaH_2_PO_4_, paraformaldehyde, citrate buffer (citric acid and sodium citrate tribasic dehydrate).

#### Enzyme-linked immunosorbent assay (ELISA)

Tissue homogenization buffer contained Triton X-100 (1%), urea (6 M), NaCl (100 mM), SDS (1%), Trizma base (50 mM), HEPES (50 mM), glycerol (10%), EDTA (1 mM), 2-mercaptoethanol (5 mM), protease inhibitor cocktail (1%) and phosphatase inhibitor cocktail (1%).

### Experimental design

Male Sprague–Dawley rats (500 g ± 150 g) were used to conduct this study. Anaesthetized (isoflurane 3.0% or medetomidine 0.5 mg/kg, and ketamine 30 mg/kg, I.P.) animals were injected in the tail vein with either saline (2 mL/kg; serving as control) or the cytostatic drug 5-FU (100 mg/kg; 50 mg/mL in saline) to induce OM. Prior to the injections, the oral mucosa of the rats was either cooled to a specific target temperature (15–30 °C) or left at intrinsic temperature (approx. 35 °C). Briefly, a portable thermostat unit (Cooral^®^ System, BrainCool AB, Lund, Sweden) [14, 16] was used to achieve the desirable temperatures. The thermostat unit was connected to a tubing system which led cooled water into a bundle of tubes which were placed in and around the oral cavity of the anaesthetized rats. A marginally invasive digital thermometer (Center 306, Sagitta Pedagog AB, Mariestad, Sweden) was used to measure the temperatures achieved by the cooling procedure.

**Fig. 1:**
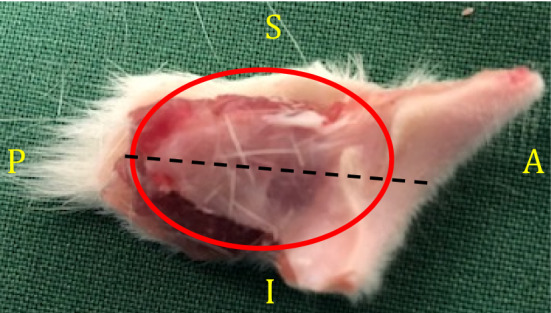
Excised rat buccal mucosa. Depiction of excised tissues utilized in the current study for histochemical and immunohistochemical (left buccal mucosa) analysis; and ELISA (right buccal mucosa). The red circle indicates the area of interest for histological analysis. The dashed black line indicates the orientation of the tissue sectioning. A Anterior; P posterior; S superior; I inferior

The animals were monitored for 1 h following tail injection of the cytostatic drug, while cooling the oral cavity to the designated temperature (15 ± 2 °C; 20 ± 2 °C; 25 ± 2 °C; 30 ± 2 °C). If necessary, the temperature was manually adjusted for the purpose to stay within the assigned temperature limits. One hour post-injection, the cooling tubes were dismounted, and the animals were injected with buprenorphine (10 μg/kg, S.C.; 0.3 mg/mL; for analgesic purposes). The rats were continuously monitored and kept on a heating pad throughout the wake-up period and were subsequently transferred to their housing cages. Three or five days after treatment, the animals were euthanized with an overdose of pentobarbital (120 mg/kg, I.P.). The left buccal mucosa (Fig. 1) was excised and stored in phosphate paraformaldehyde solution (4%; pH 7.4) for 1 day. The fixed tissue was then further dissected and stored in phosphate sucrose solution (25%; pH 7.4). Sucrose-immersed samples were sent to a company specialized in tissue sectioning (Histocenter AB, Gothenburg, Sweden) where they were embedded in paraffin and sectioned into 8 μm transversal sections. Hematoxylin and eosin (HE)-stained sections were run in a Leica Autostainer XL programmed for HE-staining according to a standard protocol. In short, the sections were deparaffinized, rehydrated, stained with hematoxylin for 6 min, followed by eosin staining for 1 min, dehydrated, and subsequently mounted with coverslips and Pertex mounting medium. The right buccal mucosa (Fig. 1) was excised, snap frozen in liquid nitrogen, and later stored in a − 80 °C freezer prior to ELISA.

### Immunohistochemistry

The left buccal mucosal sections were deparaffinized in xylene, rehydrated stepwise in solutions containing high ethanol concentration to high milli-Q water concentration and then immersed in citrate buffer, which was heated to boiling temperature (95–100 °C) in a microwave oven for 10 min. The sections were kept in the citrate solution in room temperature for 20 min. When the solution reached 40 °C, the sections were removed from the solution, followed by 10 min incubation in a solution containing copper sulphate (5 mM) and ammonium acetate (50 mM; pH 5.0). Nonspecific background staining was blocked by incubation in PBS containing 5% normal goat serum and 0.1% Triton X-100 and subsequently the sections were incubated at 4 °C overnight in PBS containing 1% normal goat serum, 0.1% Triton X-100 and a cocktail of primary antibodies; monoclonal mouse anti IL-6 (1:500; ab9324; Abcam, Cambridge, UK) and polyclonal rabbit anti TNF-α (1:500; ARC3012; Invitrogen). The following day, the sections were incubated at room temperature for 1 h in a PBS cocktail containing secondary antibodies Alexa fluor 488 goat anti-rabbit (1:500; ab150077; Abcam) and Texas Red goat anti-mouse (1:500; ab6787; Abcam) together with 1% normal goat serum and 0.1% Triton X-100. Finally, the sections were dehydrated stepwise in solutions containing high milli-Q water concentration to high ethanol concentration and mounted with coverslips and prolong gold antifade reagent containing DAPI (Abcam). All immunostainings were visualized using a Nikon 90i bright-field and fluorescence microscope, respectively, with appropriate filters for FITC, Texas Red and DAPI, fitted with a DS-Fi camera and the NIS element imaging Software v.4.40 (Nikon Corporation, Tokyo, Japan). To compare the staining intensities between the treatment groups, all images were captured using the same settings, including exposure time, contrast settings and digital gain.

### ELISA

ELISA kits were utilized for quantitative analysis of rat IL-6 (ab100785, Abcam) and rat TNF-α expression (ab100772, Abcam). The protocol provided for each kit was followed precisely. Briefly, the snap frozen tissue samples were thawed in homogenization buffer under agitation for 1 h, homogenized with an electric homogenizer and subsequently centrifuged. The supernatant was aliquoted to sterile tubes and stored at − 80 °C. The samples were analyzed for protein concentration, using Pierce BCA protein Assay Kit (Thermo Scientific, Waltham, Massachusetts, USA), in conjunction with the ELISA analysis. For the analysis, all samples were first incubated for 2.5 h at room temperature with either IL-6 or TNF-α detection antibody. A standard curve was generated from the sample recordings and the cytokine concentration was quantified. The resulting cytokine concentrations were normalized against the protein concentrations of the corresponding samples. Protein concentration and ELISA were measured with Multiscan GO (Thermo Scientific, Waltham, Massachusetts, USA).

### Data analysis

Image analysis was performed both quantitatively and qualitatively. The quantitative analysis, to measure the thickness of the mucosa, was performed using the NIS element imaging Software v.4.40 (Nikon Corporation, Tokyo, Japan) by a blinded operator. All images were obtained and analyzed in a similar fashion. IHC images were analyzed qualitatively for mucosal presence of IL-6 and TNF-α. IL-6 and TNF-α were then further analyzed quantitatively using ELISA. Power analysis was performed in G*Power software (Heinrich-Heine-Universität Düsseldorf, Düsseldorf, Germany; http://www.gpower.hhu.de/). All values are expressed as mean ± S.E.M. Statistical significance was determined by one‐way analysis of variance (ANOVA) followed by a Bonferroni correction for multiple comparisons. *P* values ≤ 0.05 were regarded as statistically significant. All statistical calculations were performed in the GraphPad Prism 8 software (GraphPad Software Inc., San Diego, CA, USA).

## Results

**Fig. 2:**
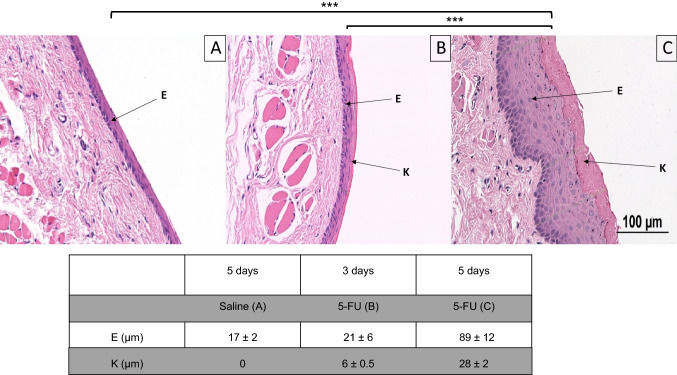
Mucosal thickness after 5-fluorouracil (5-FU) treatment. Representative images showing (**A**; *n* = 4) the absence of a keratinized outer layer in the oral epithelium of saline-treated (control) rats. Three days after treatment with 5-FU (**B**; *n* = 4) the oral epithelium displays similar thickness as compared to controls, but a distinct keratinized outer layer is visible. Five days after treatment with 5-FU (**C**; *n* = 4) the epithelium is vastly hypertrophic/hyperplastic, and a thick keratinized layer is present. *E* epithelium, *K* keratinized layer. ***p ≤ 0.001. Scale bar = 100 μm

In total, 32 Sprague–Dawley rats were used to conduct this study. Of these, 12 (37.5%) were used as part of method evaluation. When the mucosal thickness was assessed in saline-treated (control) rats five days following treatment, a combined HE stain revealed an oral epithelium with a thickness of 17 ± 2 µm, completely lacking an outer keratinized layer (Fig. 2A; *n* = 4). Three days after treatment with 5-FU, the oral epithelium was of similar thickness (21 ± 6 µm), with a thin (6 ± 0.5 µm) keratinized outer layer present (Fig. 2B; *n* = 4). Five days following treatment with 5-FU, a hypertrophic/hyperplastic oral epithelium (89 ± 12 µm) with a thickened keratinized layer (28 ± 2 µm) was visible (Fig. 2C; *n* = 4). At this time point, a statistically significant difference with regards to both epithelial thickness and keratin layer formation was observed when compared to saline-treated controls (*p* < 0.001), and 5-FU-treated oral mucosa 3 days after treatment (*p* < 0.001). However, no statistically significant difference was observed between saline-treated controls and 5-FU-treated oral mucosa 3 days after treatment (Fig. 2A, C).

**Fig. 3:**
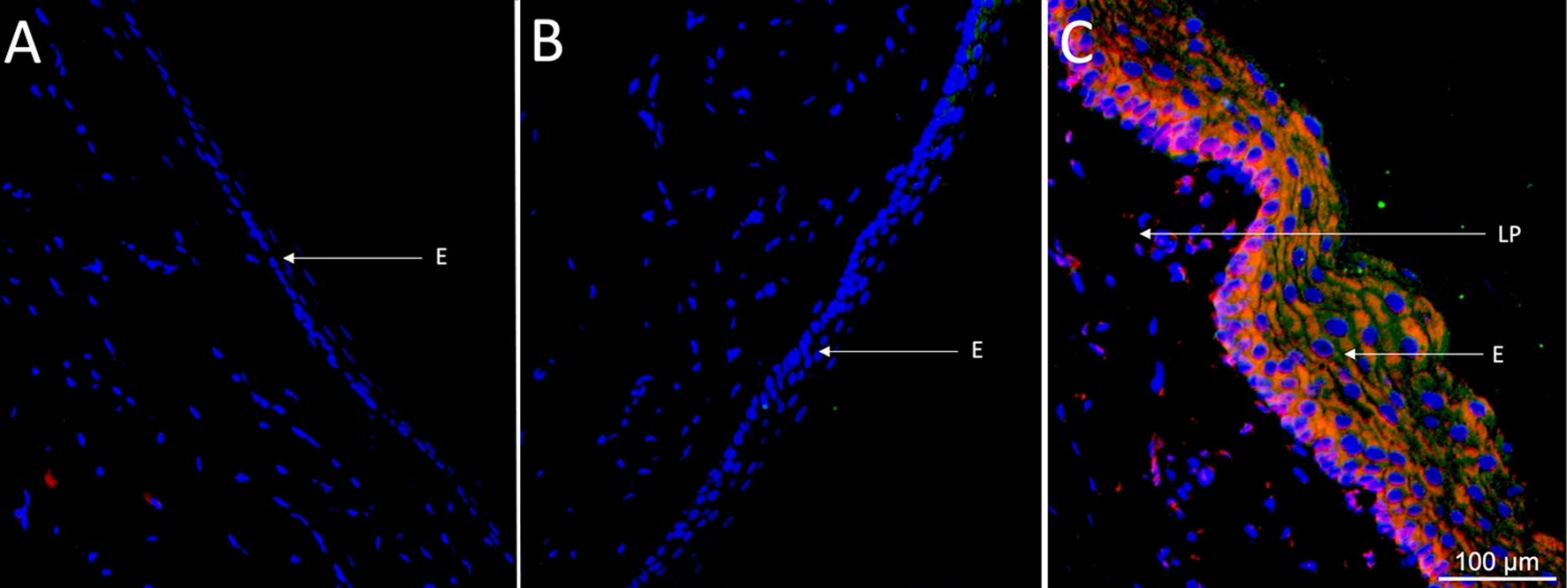
Immunohistochemical expression of IL-6 and TNF-α after 5-fluorouracil (5-FU) treatment. Representative images showing (**A**) absence of expression of IL-6 and TNF-α in the oral epithelium of saline-treated (control) rats. (**B**) Three days after treatment with 5-FU there was still no detectable expression of IL-6 or TNF-α. (**C**) Five days after treatment with 5-FU, expression of both IL-6 and TNF-α was seen in the hypertrophic/hyperplastic epithelium. Red = IL-6. Green = TNF-α. Blue = nucleus stain with DAPI. *E* epithelium. *LP* lamina propria. Scale bar = 100 μm

Concomitant immunohistochemical analysis for proinflammatory cytokine expression revealed that the oral epithelium in control tissues displayed little or no expression of IL-6 or TNF-α (Fig. 3A). Three days post-treatment with 5-FU there was still no detectable expression of neither cytokine (Fig. 3B). Five days after treatment with 5-FU, in a hypertrophic/hyperplastic state, the oral epithelium displayed distinct expression of both IL-6 and TNF-α (Fig. 3C). IL-6 was further expressed in the subepithelial tissue, i.e., the lamina propria (Fig. 3C).

**Fig. 4:**
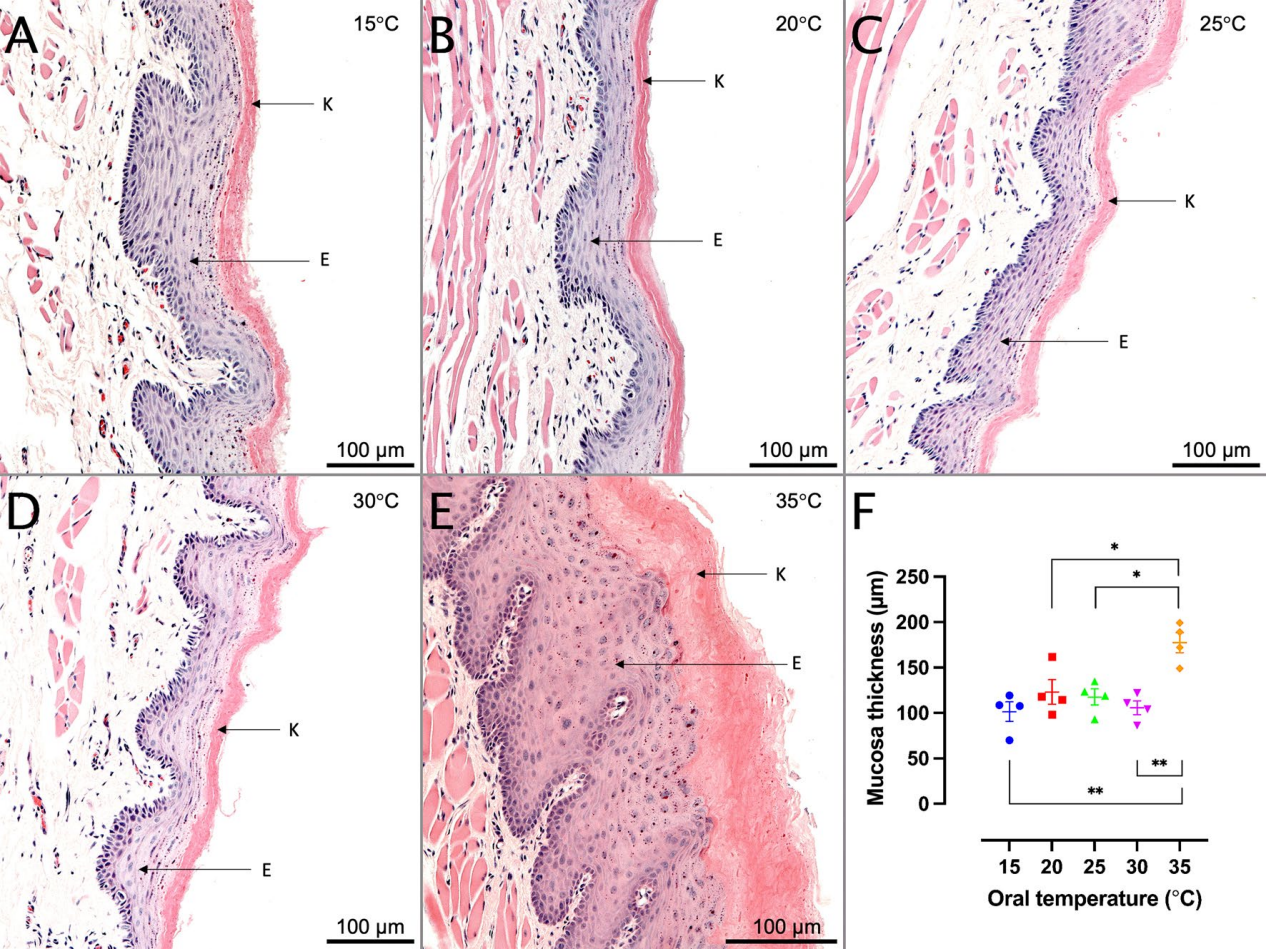
Mucosal thickness five days after 5-fluorouracil (5-FU) treatment administered in conjunction with cryotherapy. (**A**–**E**) Representative micrographs showing histological slices of the oral mucosa cooled to (**A**; *n* = 4) 15 °C; (**B**; *n* = 4) 20 °C; (**C**; *n* = 4) 25 °C; (**D**; *n* = 4) 30 °C; or (**E**; *n* = 4) 35 °C preceding to and during the 5-FU treatment. (**F**) Scatter plot depicting the cryotherapeutic effects from cooling the oral cavity to 15 °C (blue), 20 °C (red), 25 °C (green), 30 °C (purple), and control group (35 °C, orange). Cryo-treated groups (15–30 °C) displayed statistically significantly thinner mucosa as compared to the control group (35 °C), irrespective of the set temperature. There were no statistically significant differences between any of the cryo-treated groups. Data were analyzed by one-way ANOVA with Bonferroni correction. *E* epithelium, *K* keratinized layer. * *p* ≤ 0.05; ** *p* ≤ 0.01; scale bar = 100 µm

In contrast, cooling of the oral mucosa during 5-FU treatment prohibited mucosal thickening from occurring. In fact, 5 days post-5-FU treatment, the mucosa was thinner in all cryo-treated groups as compared to controls (15 °C, 101 ± 11 µm, Fig. 4A, *n* = 4; 20 °C, 123 ± 14 µm, Fig. 4B, *n* = 4; 25 °C, 118 ± 9 µm, Fig. 4C, *n* = 4; 30 °C, 106 ± 8 µm, Fig. 4D, *n* = 4; at 35 °C, 178 ± 11 µm, Fig. 4E, *n* = 4). The cryo-treated groups (15–30 °C) displayed statistically significantly thinner mucosa as compared to the control group (35 °C), irrespective of the set temperature. There were no statistically significant differences between any of the cryo-treated groups.

**Fig. 5:**
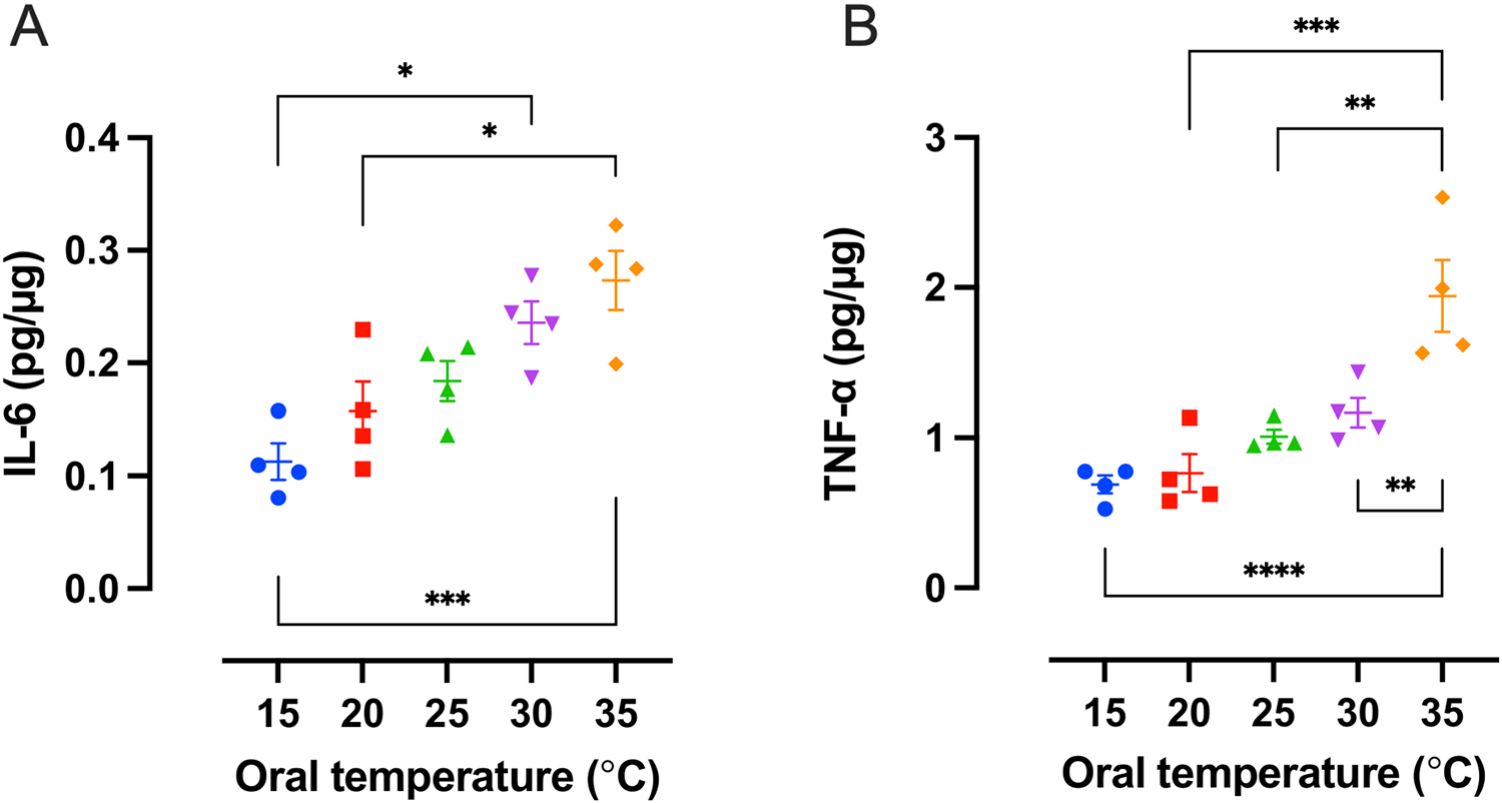
Effect of cryotherapy on the expression of IL-6 and TNF-α after 5-fluorouracil (5-FU) treatment. All measurements were conducted in tissues that were excised five days post-5-FU treatment. Cytokine expression was measured by ELISA. The expression of (**A**) IL-6 and (**B**) TNF-α was significantly decreased in tissues from animals with a cooled oral mucosa during 5-FU treatment. *p ≤ 0.05; **p ≤ 0.01; ***p ≤ 0.001; ****p  ≤ 0.0001

When the effect of cooling on the expression of the proinflammatory cytokines (IL-6 and TNF-α) was assessed, the quantitative Enzyme-linked immunosorbent assays (ELISA) showed temperature dependent increase in expression of IL-6 and TNF-α in tissues that were treated with 5-FU in absence (35 °C) or presence (15–30 °C) of the oral cooling device. The highest expression of cytokines was detected in non-cooled tissues (0.27 ± 0.03 pg/µg IL-6 and 1.94 ± 0.24 pg/µg TNF-α at 35 °C; Fig. 5A, B).

Cooling of the oral mucosa of 5-FU-treated rats to 15–20 °C counteracted the increased expression of IL-6 (0.11 ± 0.02 pg/µg vs 0.27 ± 0.03 pg/µg at 15 °C and 35 °C, respectively; *p* = 0.0009; 0.16 ± 0.03 pg/µg vs 0.27 ± 0.03 pg/µg at 20 °C and 35 °C, respectively; *p* = 0.0172; Fig. 5A). Also, a difference in IL-6 expression was shown between two cooling groups (0.11 ± 0.02 pg/µg vs 0.24 ± 0.02 pg/µg at 15 °C, and 30 °C, respectively; *p* = 0.0172; Fig. 5A). Similarly, cooling of the oral mucosa to 15–30 °C counteracted the increase of TNF-α (0.69 ± 0.06 pg/µg vs 1.94 ± 0.24 pg/µg at 15 °C and 35 °C, respectively; *p* < 0.0001; 0.77 ± 0.13 pg/µg vs 1.94 ± 0.24 pg/µg at 20 °C and 35 °C, respectively; *p* = 0.0002; 1.01 ± 0.05 pg/µg vs 1.94 ± 0.24 pg/µg at 25 °C, and 35 °C, respectively; *p* = 0.0016; 1.17 ± 0.12 pg/µg vs 1.94 ± 0.24 pg/µg at 30 °C, and 35 °C, respectively; *p* = 0.0087; Fig. 5B).

## Discussion

The current study shows that a single intravenous (*i.v.*) injection with a clinically relevant dose of the cytostatic drug 5-FU leads to hypertrophy/hyperplasia in the rat oral epithelium and an increased keratinized layer five days following treatment. Further, the treatment induces an inflammatory state, which was demonstrated by an increased expression of the proinflammatory cytokines IL-6 and TNF-α. The hypertrophy/hyperplasia of the oral epithelium together with an increased expression of IL-6 and TNF-α resembles the early events of OM, which have previously been described in a contemporary model of mucositis development [7]. Furthermore, our results are consistent with previous studies which have demonstrated that 5-FU causes OM in rats [17, 18], similar to the clinical situation in patients following antineoplastic treatment. It should be noted that the occurrence of OM is most likely dose dependent. At lower concentrations of the cytotoxic drug, the oral epithelium will respond with a cytokine-driven proliferation, but at higher concentrations a cytokine storm will cause an ulceration. This could explain why no signs of ulceration in oral mucosa are currently observed. Nevertheless, the current single injection of 5-FU clearly gave rise to proliferative effects and a proinflammatory response in the rat oral epithelium.

It is well known that IL-6 and TNF-α can act as proinflammatory mediators and several studies have shown that IL-6 and TNF-α often act in concordance, in particular in the oral mucosa [19, 20]. Likewise, both IL-6 and TNF-α have been shown to be expressed in human oral keratinocytes [21]. Apart from being proinflammatory, several studies have shown that IL-6 stimulates various cell types to proliferate, including fibroblasts and keratinocytes [22, 23]. It is, therefore, likely that IL-6, and probably also TNF-α, are at least partly responsible for the observed hypertrophy/hyperplasia of the oral epithelium with subsequent keratinization.

To the best of our knowledge, this is the first study that examines effects of cryotherapy (CT) on early events of chemotherapy-induced OM in rats. The data show clear beneficial effects of CT, with complete absence of epithelial thickening already at a moderate cooling temperature (30 °C). Previous studies aiming to identify optimal temperatures for CT after cardiac arrest or traumatic brain injury have not been able to show advantages of considerably decreased temperatures [24, 25]. On the contrary, a mild to moderately decreased temperature seems to be best tolerated and yield best long-term outcomes. Other animal models have shown that hypothermia can reduce the injury area and have direct effects on levels of IL-1β after traumatic brain injury [26], thereby indicating the beneficial effects of CT to be at least partly due to decrease of expression of inflammatory mediators. Currently, further cooling, beyond 30 °C, seems beneficial regarding the expression of proinflammatory cytokines. However, since no evident signs of inflammation could be observed in the histological samples already after moderate cooling, it is difficult to distinguish if further reduction of expression of IL-1β and TNF-α at lower temperatures has additional advantageous effects.

It should be noted that qualitative assessment of immunohistochemical images has certain limitations. One such is the risk of biased analysis. However, careful measurements, including blinding, were undertaken to avoid such pitfalls. Also, all qualitative analyses are currently correlated to quantitative data, i.e., mucosal thickness and protein expression (ELISA).

Combined with existing evidence, the results of the present study confirm that CT is an effective strategy to prevent the events which may precede clinically established OM. Furthermore, suggesting that cooling at higher temperatures, e.g., using cooling devices that can operate at higher temperatures [12, 14], may be advantageous in clinical settings to prevent chemotherapy-induced OM.

## Conclusion

The findings in this study show that a bolus *i.v.* injection with 5-FU in rats can be used to create a functional animal model for chemotherapy-induced OM with signs of thickening and increased keratinization of the oral mucosa and expression of proinflammatory cytokines. Further, this study demonstrates the usefulness of oral mucosal cooling to prevent the early events which may precede clinically established OM. From a clinical point of view, it is of interest that the current data indicates that cooling to low temperatures (< 30 °C), which causes discomfort for the patient, may not be necessary to achieve the desired outcome.

## Data Availability

The datasets generated during and/or analysed during the current study are available from the corresponding author on reasonable request.

## Abbreviations

5-FU: 5-Fluorouracil
CT: Cryotherapy
ELISA: Enzyme-linked immunosorbent assay
HE: Hematoxylin and eosin
I.P: Intraperitoneal
I.V: Intravenous
IHC: Immunohistochemistry
IL-6: Interleukin-6
OM: Oral mucositis
S.C.: Subcutaneous
TNF-α: Tumor necrosis factor alpha
